# Effect of football activity and physical fitness on information processing, inhibitory control and working memory in adolescents

**DOI:** 10.1186/s12889-020-09484-w

**Published:** 2020-09-14

**Authors:** Ryan A. Williams, Simon B. Cooper, Karah J. Dring, Lorna Hatch, John G. Morris, Caroline Sunderland, Mary E. Nevill

**Affiliations:** Department of Sport Science; Exercise and Health Research Group; Sport, Health and Performance Enhancement (SHAPE) Research Centre, Nottingham Trent University, Nottingham, UK

**Keywords:** Exercise, Physical activity, Cognition, Executive function, Working memory, Soccer, Football

## Abstract

**Background:**

Whilst an acute bout of exercise has been shown to enhance subsequent cognition, including in adolescents, the effects of team games (of which Football is the most popular) has received little attention. Therefore, this study examined: the effect of an acute bout of outdoor Football activity on information processing, inhibitory control, working memory and circulating brain-derived neurotrophic factor (BDNF) in adolescents; the effect of physical fitness on cognition and; the moderating effect of physical fitness on the acute exercise responses.

**Methods:**

Following familiarisation, 36 adolescents (16 girls) took part in two trials (60-min Football and 60-min seated rest) separated by 7-d in a counterbalanced, crossover design. Information processing and inhibitory control (Stroop Test), and working memory (Sternberg Paradigm) were assessed 30-min before exercise/rest and immediately, 45- and 90-min post-exercise/rest. Capillary blood samples were obtained before exercise/rest and up to 120-min post-exercise/rest. The median split of distance covered on the MSFT was used to divide the group into high- and low-fit groups.

**Results:**

Performance on the cognitive function tasks was similar between Football and seated rest (trial*time interactions; all *p* > .05). However, the high-fit group had overall quicker response times on both levels of the Stroop Task and all three levels of the Sternberg Paradigm (main effect of fitness; all *p* < .001). Furthermore, the exercise-cognition relationship was moderated by physical fitness, with improvements in working memory response times seen post-exercise, only in the high-fit group (trial*time*fitness interaction, *p* < .05). Circulating BDNF was unaffected by the Football activity and physical fitness (*p* > .05).

**Conclusion:**

The present study shows that higher levels of physical fitness are beneficial for cognitive function and provides novel evidence that an ecologically valid, and popular, form of exercise is beneficial for working memory following exercise, in high-fit participants only.

## Background

Acute bouts of exercise elicit small-moderate beneficial effects on cognitive function in adults [1], children [2] and adolescents [3]. However, the exercise-cognition relationship is a complex phenomenon, affected by a number of factors such as the modality, intensity and duration of the exercise bout, age, physical fitness and the cognitive domain assessed [4, 5]. Much of the research in the adolescent population has employed traditional continuous lab-based exercise protocols, examining treadmill running/walking [6–8] and cycle ergometry [9–12]. Furthermore, the focus has been primarily on cognitive function immediately post-exercise, including the domains of executive function and working memory [6, 9, 13]. There is also some evidence that the benefits of an acute bout of exercise persist for up to 45 min [14–16] post-exercise; yet the time-course of exercise-induced cognitive effects beyond this are currently unknown. Only one study has demonstrated acute cognitive benefits up to 60 min post-exercise, with improved inhibitory control found 60 min following moderate intensity circuit exercise [17]. However, no study has examined the effect of an acute bout of exercise beyond 1 h post-exercise in adolescents.

Furthermore, many of the acute exercise protocols used in previous studies are difficult to incorporate into a school day due to reliance on specialist equipment, such as motorised treadmills and cycle ergometers, which may not be available in a school setting. This is known to be a prominent barrier to exercise participation in this population [18]. Recent research has attempted to address this issue by utilising acute school-based protocols consisting of sprint intervals [14], shuttle running [15, 19], Basketball [16] and cognitively-engaging exercise [13, 20]. The use of acute games-based activity, such as Football, is an attractive modality given that the habitual activity patterns of young people are high-intensity and intermittent in nature [21], as seen in team games [22]. Furthermore, games-based activity is typically a mode of exercise that young people enjoy; a vital consideration for long-term implementation [23]. Positive effects of such acute school-based protocols have been demonstrated across a range of domains of cognition, including attention [13, 15, 20], working memory [16] and executive function [14, 16]. Football is the most popular games-based exercise amongst adolescents [23], with only one study to date examining the acute effects of Football on subsequent cognitive function [24]. A brief (20 min) bout of high-intensity Football improved inhibitory control performance 20 min post-exercise, compared to walking Football and a resting control [24]. However, it is unknown how acute Football affects other domains of cognitive function, such as working memory, as well as the duration of the transient improvements post-exercise.

Cross-sectional evidence in adults suggests that those with a higher physical fitness, assessed by V̇O_2_max, have quicker response times on a psychomotor speed task [25]. Similar results have been demonstrated in overweight and sedentary children, using V̇O_2_ peak as the fitness criterion [26]. Hillman et al. [27] found that both high-fit children and adults, assessed with the PACER, a variation of the multi-stage fitness test, performed better on an executive function task than their low-fit counterparts. Whilst there is strong evidence of a positive relationship between physical fitness and cognitive function in children and adults, there is limited knowledge concerning adolescents. Adelantado-Renau et al. [28] used a battery of fitness tests, including the multi-stage fitness test, in a group of healthy adolescents and found that higher physical fitness was positively associated with academic performance. In addition, higher physical fitness (assessed by the Andersen intermittent test) was associated with a greater inhibitory control performance in older adolescents (~ 14 y) [29]. However, it is not known how physical fitness affects key cognitive domains such as executive function and working memory in younger adolescents, where they are of particular importance for academic achievement [30].

Recent reviews suggest that physical fitness moderates the acute exercise-cognition relationship [1, 4] – particularly when cognition is measured immediately post-exercise [1], or with reference to learning and memory [4]. However, another recent meta-analysis concluded that physical fitness does not moderate the acute exercise response, with respect to aerobic exercise and executive function [3]. Specifically, it has been shown that adolescents with a higher level of physical fitness, assessed by a multi-stage fitness test [16] and a continuous-graded maximal exercise test until exhaustion [11], demonstrate improved response times on an executive function task immediately after cycling [11] and 45 min after basketball exercise [16]; whilst in lower fit adolescents error rates were higher [11] and response times were slower [16] following exercise. In addition, response times on a working memory task were improved, in the high-fit group only, following basketball exercise [16]. Overall, the available evidence suggests that higher physical fitness may enhance the post-exercise improvements in cognition. This may also be more applicable to games-based exercise, which has both cognitive and physical demands, whereby those with a higher physical fitness can allocate greater cognitive resources to the activity itself [3]. The underlying mechanisms behind this relationship remain unclear, although it has been surmised that circulating growth factors – particularly brain-derived neurotrophic factor (BDNF) – may have a role to play [31, 32].

BDNF is stated to have an instrumental role in the structural formation and function of the brain [33] and plays an important role in the promotion and maintenance of synaptic connectivity [34]; which is suggested to be one of the mechanisms through which BDNF may mediate post-exercise improvements in cognitive function [4]. To date, only resting BDNF has been investigated in relation to objectively measured physical activity in adolescents; whereby physical activity and plasma BDNF were not related [35] and mean physical activity and serum BDNF were negatively related in adolescent boys only [36]. Furthermore, no studies have examined the time-course of BDNF concentrations in the hours following an acute bout of exercise in adolescents – which is an important knowledge gap to fill given the suggested role of BDNF in mediating post-exercise cognitive improvements [31, 33]. The response of BDNF to an acute bout of ecologically valid games-based activity in adolescents, and the moderating role of physical fitness in this exercise-BDNF relationship, is currently unknown.

The aim of the present study was to investigate the effect of an acute bout of outdoor Football on information processing, inhibitory control and working memory and circulating BDNF concentration in adolescents, for up to 2 h post-exercise. A secondary aim of the study was to examine whether there were differences in overall cognitive function performance and BDNF concentration between high and low-fit participants, and whether physical fitness moderates cognitive function and BDNF concentrations following exercise.

## Methods

### Participant characteristics

Thirty-six adolescents (20 boys, 16 girls) volunteered to participate in the study. During familiarisation, all participants underwent anthropometric measurements of height (cm), body mass (kg) and sitting height (cm). These were used to calculate age at peak height velocity, using previously described methods [37]. Height was measured with a Leicester Height Measure (Seca, Hamburg, Germany) accurate to 0.1 cm, body mass was measured using a Seca 770 digital scale (Seca, Hamburg, Germany) accurate to 0.1 kg and waist circumference to the nearest 0.1 cm [38]. Four skinfold sites were measured (triceps, subscapular, supraspinale and front thigh) as a surrogate marker of body composition, in line with previously described methods [39]. Descriptive participant characteristics are presented in Table 1.

**Table 1:** Participant characteristics for the group overall, as well as for the high- and low-fit groups. Data are mean ± SD

Variable	Overall	High-fit	Low-fit
*Age (yrs)*	12.6 ± 0.5	12.7 ± 0.5	12.4 ± 0.5
*Height (cm)*	163.1 ± 7.0	163.5 ± 8.0	162.6 ± 6.1
*Body Mass (kg)*	53.9 ± 10.0	50.1 ± 8.9 *	57.6 ± 10.0
*Waist Circumference (cm)*	70.0 ± 8.0	66.0 ± 5.4 *	73.9 ± 8.4
*Sum of 4 skinfolds (mm)*	60.3 ± 26.8	42.8 ± 12.8	77.8 ± 25.7
*Maturity Offset*	0.08 ± 0.94	0.20 ± 0.84	−0.05 ± 1.03
*MSFT Distance (m)*	1160 ± 400	1480 ± 300 **	840 ± 140
*Predicted V̇O*_*2*_ *peak (ml·kg*^*−1*^*·min*^*−1*^*)* ^*a*^	47.9 ± 5.2	52.0 ± 3.7 **	43.8 ± 2.5

Abbreviations: *MSFT* Multi-Stage Fitness Test

^a^ predicted from the multi-stage fitness test using the equations of Barnett et al [40]

* Different compared to low-fit, *p* < .05. ** Different compared to low-fit, *p* < .001

### Experimental design

The study conformed to the Declaration of Helsinki guidelines and was approved by the institution’s Human Ethics Committee. Participants were recruited from secondary schools in the East Midlands area of the UK. Written parental consent and participant assent were obtained during the initial phase of recruitment. A health screen was completed by each participant’s parent/guardian and was checked by a lead investigator to ensure there were no medical conditions that would affect the child’s participation in the study. All participants that enrolled in the study were considered healthy. In particular, any participants that had existing neurological and/or mental health conditions were not eligible to take part. In addition to this, participants with any physical ailments that could be exacerbated by physical activity were not permitted to take part.

This study employed a randomised, order-balanced, crossover, within-subjects design consisting of two main experimental trials (exercise and resting); separated by at least 7 d. Participants were blind to the trial condition until arrival at school. A familiarisation took place ~ 7 d before the first main trial and allowed participants to be acquainted with all the necessary procedures, including capillary blood sampling and the battery of cognitive function tests. Participants were also familiarised with a Football session, consisting of skill drills and small-sided games to ensure they had the required skills to participate in the Football session as part of the exercise trial.

During the familiarisation, participants also completed the multi-stage fitness test (MSFT) [41] for the assessment of physical fitness. Prior to the start of the MSFT, participants were fitted with a heart rate monitor (Firstbeat Team Sport System, Firstbeat Technologies Ltd., Finland). Heart rate was monitored throughout the MSFT and maximum heart rate was recorded upon completion. To encourage maximum effort from the participants, investigators provided verbal encouragement. Performance on the test was determined by the total distance covered (m), with participants assigned to high- and low-fitness groups, based on the median split of the MSFT distance covered for each sex.

#### Main trials

Participants were instructed to record their dietary intake for the 24 h preceding and during the first experimental trial. Recorded diets were then replicated for the subsequent main trial. Participants were asked to refrain from eating or drinking from 9 pm the previous evening for both days of the two main trials. Water was allowed ad libitum at all times. Participants were also asked to refrain from any unusually strenuous physical activity 24 h prior to the main trials. Parents/guardians were contacted by telephone on the evening prior to each main trial to ensure compliance with these requirements.

On the morning of the main trials, following the overnight, fast participants reported to school (between 8 am and 8:30 am) and were fitted with a heart rate monitor (Firstbeat Team Sport System, Firstbeat Technologies Ltd., Finland). Both trials followed a time matched protocol, with the only difference being the 60 min exercise session. During the exercise trial participants completed 60 min Football (see section 2.3.4); whilst they remained seated in the classroom during the resting control trial. In both trials, participants were allowed to interact freely with each other.

### Experimental procedures

#### Standardised breakfast and lunch

In order to better ascertain the sole effects of exercise, a standardised breakfast and lunch was provided for participants during the experimental trials. The breakfast provided 1.5 g of carbohydrate per kg body mass (cornflakes, milk, white toast and butter). The lunch also provided 1.5 g of carbohydrate per kg body mass (chicken sandwich, baked crisps and an apple; with a cheese alternative for vegetarians). Both of these meals were used to control for the influence of nutrition on cognitive function [42], the potential for nutrition and exercise to interact to affect cognition [43] and were exactly the same for both trials. Participants were given 15 min to consume each meal. All participants complied with this requirement.

#### Capillary blood samples

Capillary blood samples were preferred over venous samples due to ethical constraints and have also been used successfully in an adolescent population [39]. Capillary blood samples were taken at baseline and immediately, 30 min and 60 min post-exercise. An additional blood sample was taken 60 min post-lunch (2 h post-exercise).

In order to increase capillary blood flow, participants’ hands were warmed via submersion in warm water prior to collection. A Unistik single-use lancet (Unistik, Extra, 21G gauge, 2.0 mm depth, Owen Mumford Ltd., UK) was used and blood was collected into a single 300 μl microvette, with clotting activator (Microvette CB 300 Z, Sarstedt Ltd., UK). The sample was allowed to rest for 30 min at room temperature before undergoing centrifugation at 1000 x g for 15 min (Eppendorph 5415C, Hamburg, Germany). Serum was then extracted into 500 μl plastic vials for subsequent analysis. All samples were frozen immediately at − 20 °C and transferred to − 80 °C as soon as possible. Brain-derived neurotrophic factor (BDNF) concentrations were determined with a commercially available ELISA (Quantikine ELISA®, R & D Systems Europe Ltd., UK) according to the manufacturer’s instructions. The intra-assay coefficient of variation (%) for 8 repeat measurements was 6.9%.

#### Cognitive function tests

The cognitive function test battery lasted approximately 8 min and consisted of the Stroop test and the Sternberg Paradigm, completed in this order on a laptop computer (Lenovo ThinkPad T450; Lenovo, Hong Kong). Each test and test level were preceded by instructions on the screen and practice stimuli in order to re-familiarise participants with the test and negate any potential learning effects; the data for the practice stimuli were discarded. The participants completed the tests in a classroom of 10 participants, in silence and separated so that they could not interact during the tests. Participants were seated 80–100 cm from the screen in a self-selected position that was comfortable. Sound cancelling headphones were worn and the lights in the room dimmed to minimise external disturbances and enhance screen visibility. For each test the variables of interest were the response times (ms) of correct responses (i.e. reaction time + movement time) and the proportion (%) of correct responses made. For a visual representation of the cognitive testing procedures, please see Supplementary Fig. 1.

##### Stroop test

The Stroop test measures information processing and executive function (in particular the domain of inhibitory control) [44]. The Stroop test consisted of two levels (congruent and incongruent). During both levels, a test word was placed in the centre of the screen with a target and distractor placed randomly on the left and right side. Participants were instructed to select their response using the appropriate arrow keys (left or right) with the index and middle fingers of the right hand respectively. On the congruent level, there were 20 stimuli with the test, target and distractor stimuli all presented in white ink. The incongruent level (colour interference) contained 40 stimuli, with participants selecting the ink colour that the test word was displayed in, rather than the word itself (e.g. if ‘red’ was written in green font, the correct response would be green). For both levels, participants were instructed to respond as quickly and as accurately as possible. Choices remained on screen until participants responded, with an inter-stimulus interval of 1 s.

##### Sternberg paradigm

The Sternberg paradigm measures the domain of working memory [45] and consisted of three levels of ascending complexity. Each level used a different working memory load (one, three or five items). The one item level always used the number ‘3’ as the target and consisted of 16 test stimuli. The three and five item levels had targets that were randomly generated (e.g. three-item ‘A T W’; five-item: ‘B G N I R’), with each level containing 32 test stimuli. At the start of each level, the target items were displayed with instructions to press the right arrow key (with the middle finger of their right hand) if a target was present, or the left arrow key (with the index finger of their right hand) for a distractor. The correct response was counterbalanced between the left and right arrow key for each level. On all levels the choice stimuli were presented in the centre of the screen, with an inter-stimulus interval of 1 s.

#### Exercise protocol

The exercise consisted of a 60 min Football session. Football was chosen because it is high-intensity and intermittent in nature and thus replicates the activity patterns typically observed in this population [21]; as well as being an enjoyable and popular form of games-based activity for young people [46] and thus has ecological validity. A duration of 60 min was selected to advance on previous work examining the effects of 60 min of Basketball [16], whereas research utilising Football protocols is typically shorter in duration (20 min) [24]. An experienced Football coach delivered the sessions to groups of 10 participants, on outdoor facilities at the respective schools. The session consisted of a warm-up (5 min), skill-based drills (25 min) and small-sided games (5 vs 5; 30 min). Heart rate was monitored continuously throughout the session. Maximum heart rate (HRmax), as recorded at the end of the MSFT, and heart rate during the Football session were used to calculate the relative exercise intensity (%HRmax). Additionally, Global Positioning System (GPS) devices were worn to quantify the external load during the Football session using SPI HPU (15 Hz) portable GPS units (GPSports, Australia). The GPS units were fitted to sit between the scapulae, at the base of the cervical spine, using an elasticated shoulder harness. After each exercise session, the data were downloaded to Team AMS software (Team AMS, GPSports). Variables of interest are expressed as total distance covered (m) as well as distance covered at low-speed (< 9 km·h^− 1^), moderate-speed (9–13 km·h^− 1^) and high-speed (> 13 km·h^− 1^) [47].

### Statistical analysis

Data from the Stroop test and Sternberg paradigm were analysed using the open source software, R (www.r-project.org). Response time and accuracy analyses were conducted using Analysis of Covariance (ANCOVA), with waist circumference as a covariate – given the effect of waist circumference on executive function [48]. Prior to analyses, the response times (of correct responses) were log transformed to exhibit the right hand skew, typical of human response times. Initially a three-way (trial by time by fitness) ANCOVA (with repeated measures for trial and time) was conducted. Where three-way interactions occurred, separate two-way (trial by time) repeated measures ANCOVAs for each fitness group were conducted. To explore statistically significant two-way interactions (trial by time), paired samples t-tests with a Bonferroni correction for multiple comparisons were conducted to compare between the trials at each time point. These analyses allow exploration of the effects of the football session, and the moderating effect of fitness, on subsequent cognitive function. For the response time analyses, minimum (< 200 ms) and maximum (1500–3000 ms, dependent on task complexity) were applied to eliminate any unreasonably fast and slow responses.

BDNF analysis conducted using SPSS (Version 25; SPSS Inc., Chicago, IL., USA), also adopting a three-way (trial by time by fitness) ANOVA (with repeated measures for trial and time). Heart rate and GPS variables were compared between the high- and low-fitness groups using an independent samples t-test. All data are presented as mean ± SD, unless otherwise stated. Alpha (α) was set at *p* < .05.

## Results

### Exercise characteristics

During the 60 min of Football, average heart rate was 151 ± 16 beats^.^min^− 1^, maximum heart rate was 186 ± 13 beats^.^min^− 1^ and relative exercise intensity was 75 ± 8% (Table 2). Furthermore, average (*t*_(32)_ = − 2.8, *p* = .009) and maximum heart rate (*t*_(32)_ = 2.7, *p* = .010), as well as relative exercise intensity (*t*_(32)_ = − 4.1, *p* < .001), were lower in the high-fit group compared to low-fit (Table 2). Average heart rate across the whole 4 h exercise trial (105 ± 13 beats^.^min^− 1^) was higher than during the whole 4 h resting trial (84 ± 11 beats^.^min^− 1^, *t*_(35)_ = 11.8, *p* < .001).

**Table 2:** Average and maximum heart rate, relative exercise intensity and GPS characteristics for the group overall, as well as the high- and low-fitness splits, during the 60 min Football session. Data are mean ± SD

Variable	Overall	High-Fit	Low-Fit
*Heart Rate*			
Average Heart Rate (beats·min^− 1^)	151 ± 16	144 ± 16 *	158 ± 12
Maximum Heart Rate (beats·min^− 1^)	186 ± 13	180 ± 11 *	191 ± 11
Relative Exercise Intensity (% maximum heart rate) ^b^	75 ± 8	70 ± 7 *	80 ± 6
*GPS*			
Total Distance (m)	2788 ± 432	2783 ± 399	2791 ± 474
Low Speed Distance (m)	2129 ± 278	2099 ± 274	2158 ± 287
Moderate Speed Distance (m)	477 ± 120	484 ± 107	469 ± 133
High Speed Distance (m)	177 ± 84	191 ± 68	161 ± 97

Abbreviations: *GPS* Global Positioning System. *MSFT* Multi-Stage Fitness Test

^b^ Relative to the maximum heart rate attained during the MSFT

* High-fit group lower than low-fit, *p* < .01

During the Football session, the average total distance covered was 2788 ± 432 m. Of this, 2129 ± 278 m was covered at low speed (< 9 km·h^− 1^), 477 ± 120 m at moderate speed (9–13 km·h^− 1^) and 177 ± 84 m at high speed (> 13 km·h^− 1^). All GPS variables during the 60 min football session were similar between the high- and low-fit groups (Total Distance; *p* = .959. Low Speed Distance; *p* = .542. Moderate Speed Distance; *p* = .710. High Speed Distance; *p* = .305, Table 2).

### Cognitive function

Data for both cognitive tests, across all time points, for the exercise and control trials are displayed in Table 3. Given that there were no differences at baseline between the exercise and resting trials (all *p* > .05) and for ease of interpretation, the figures are displayed as change from baseline. An overview of the results of the statistical analyses is displayed in supplementary Table 1.

**Table 3:** Cognitive function data across the exercise and control trials, for the high- and low-fitness groups, as well as the group overall. Data are mean ± SD

Test	Level	Variable	Group	Control Trial				Exercise Trial			
				Pre-rest	Immediately post-rest	45 min post-rest	90 min post-rest	Pre-exercise	Immediately post-exercise	45 min post-exercise	90 min post-exercise
**Stroop**	*Simple*	Response Time (ms)	Low-fit	857 ± 192	812 ± 179	753 ± 136	756 ± 144	828 ± 172	755 ± 161	807 ± 152	785 ± 175
			High-fit	772 ± 170	717 ± 141	723 ± 150	712 ± 149	746 ± 145	712 ± 78	684 ± 85	695 ± 134
			Overall	815 ± 185	764 ± 165	738 ± 141	734 ± 145	787 ± 161	733 ± 127	745 ± 139	740 ± 161
		Accuracy (%)	Low-fit	99.7 ± 1.2	95.8 ± 7.7	94.2 ± 8.4	94.7 ± 9.0	99.4 ± 1.6	96.9 ± 4.2	97.8 ± 3.1	96.7 ± 5.4
			High-fit	97.2 ± 4.0	97.8 ± 3.6	95.8 ± 5.9	96.1 ± 4.4	98.9 ± 2.6	97.8 ± 3.1	95.8 ± 6.3	95.0 ± 5.9
			Overall	98.5 ± 3.1	96.8 ± 6.0	95.0 ± 7.2	95.4 ± 7.0	99.2 ± 2.2	97.4 ± 3.7	96.8 ± 5.0	95.8 ± 5.5
	*Complex*	Response Time (ms)	Low-fit	1130 ± 232	1074 ± 236	1059 ± 219	1017 ± 285	1168 ± 226	1094 ± 291	1089 ± 242	1042 ± 223
			High-fit	1037 ± 253	939 ± 192	954 ± 202	973 ± 235	957 ± 202	950 ± 206	920 ± 185	953 ± 193
			Overall	1084 ± 243	1006 ± 224	1007 ± 216	995 ± 257	1062 ± 237	1022 ± 260	1005 ± 231	997 ± 214
		Accuracy (%)	Low-fit	96.1 ± 5.4	94.9 ± 6.7	92.7 ± 10.5	92.9 ± 10.0	96.7 ± 4.3	91.4 ± 13.5	95.3 ± 4.9	93.6 ± 11.2
			High-fit	96.3 ± 4.0	94.9 ± 4.6	94.2 ± 5.4	95.1 ± 5.0	96.3 ± 3.9	94.9 ± 5.2	93.6 ± 5.7	91.8 ± 5.4
			Overall	96.2 ± 4.6	94.9 ± 5.6	92.9 ± 8.3	94.0 ± 7.8	96.5 ± 4.0	93.1 ± 10.2	94.4 ± 5.2	92.7 ± 8.7
**Sternberg**	*One item*	Response Time (ms)	Low-fit	588 ± 164	520 ± 102	531 ± 110	480 ± 118	533 ± 135	502 ± 83	538 ± 135	535 ± 120
			High-fit	516 ± 97	486 ± 87	507 ± 120	482 ± 76	517 ± 111	473 ± 56	476 ± 85	508 ± 91
			Overall	552 ± 138	503 ± 95	519 ± 114	481 ± 98	525 ± 122	488 ± 71	507 ± 115	521 ± 106
		Accuracy (%)	Low-fit	96.5 ± 5.3	97.9 ± 4.3	95.8 ± 5.3	94.8 ± 9.9	96.2 ± 5.3	94.8 ± 7.5	95.5 ± 5.6	93.8 ± 10.9
			High-fit	97.6 ± 3.1	97.2 ± 4.9	96.9 ± 4.4	97.2 ± 4.4	97.9 ± 3.7	97.2 ± 4.4	97.2 ± 3.8	95.8 ± 5.3
			Overall	97.0 ± 4.4	97.6 ± 4.6	96.4 ± 4.8	96.0 ± 7.6	97.0 ± 4.6	96.0 ± 6.2	96.4 ± 4.8	94.8 ± 8.5
	*Three item*	Response Time (ms)	Low-fit	743 ± 185	718 ± 157	691 ± 185	697 ± 153	696 ± 118	705 ± 158	698 ± 146	674 ± 186
			High-fit	660 ± 149	621 ± 113	597 ± 100	613 ± 85	628 ± 111	631 ± 123	606 ± 98	672 ± 128
			Overall	701 ± 171	670 ± 144	644 ± 154	655 ± 129	662 ± 118	668 ± 145	652 ± 131	673 ± 157
		Accuracy (%)	Low-fit	97.1 ± 3.5	96.0 ± 5.1	93.2 ± 8.0	92.9 ± 10.0	96.2 ± 5.1	95.7 ± 6.1	95.8 ± 5.1	95.1 ± 5.1
			High-fit	97.2 ± 2.6	96.2 ± 4.7	95.3 ± 5.4	95.0 ± 4.9	95.3 ± 6.0	95.3 ± 3.8	95.7 ± 4.6	94.8 ± 5.7
			Overall	97.1 ± 3.0	96.1 ± 4.9	94.3 ± 6.8	93.9 ± 7.9	95.7 ± 5.5	95.5 ± 5.0	95.7 ± 4.8	95.0 ± 5.3
	*Five item*	Response Time (ms)	Low-fit	926 ± 205	802 ± 222	762 ± 259	787 ± 182	887 ± 172	878 ± 215	794 ± 189	838 ± 181
			High-fit	799 ± 175	813 ± 194	701 ± 154	745 ± 146	771 ± 128	741 ± 125	727 ± 118	794 ± 147
			Overall	863 ± 199	807 ± 206	732 ± 213	766 ± 164	829 ± 161	810 ± 187	761 ± 159	816 ± 164
		Accuracy (%)	Low-fit	93.8 ± 6.5	89.4 ± 11.9	86.8 ± 16.5	90.5 ± 13.9	91.8 ± 9.5	89.8 ± 9.2	88.7 ± 13.4	86.8 ± 12.8
			High-fit	94.1 ± 6.9	93.2 ± 6.5	92.2 ± 6.7	93.1 ± 7.3	93.2 ± 7.1	94.3 ± 5.8	92.7 ± 6.8	91.7 ± 6.8
			Overall	92.5 ± 8.3	92.0 ± 7.9	90.7 ± 10.6	89.2 ± 10.4	92.5 ± 8.3	92.0 ± 7.9	90.7 ± 10.6	89.2 ± 10.4

### Stroop test

#### Response times

##### Congruent level

**Fig. 1:**
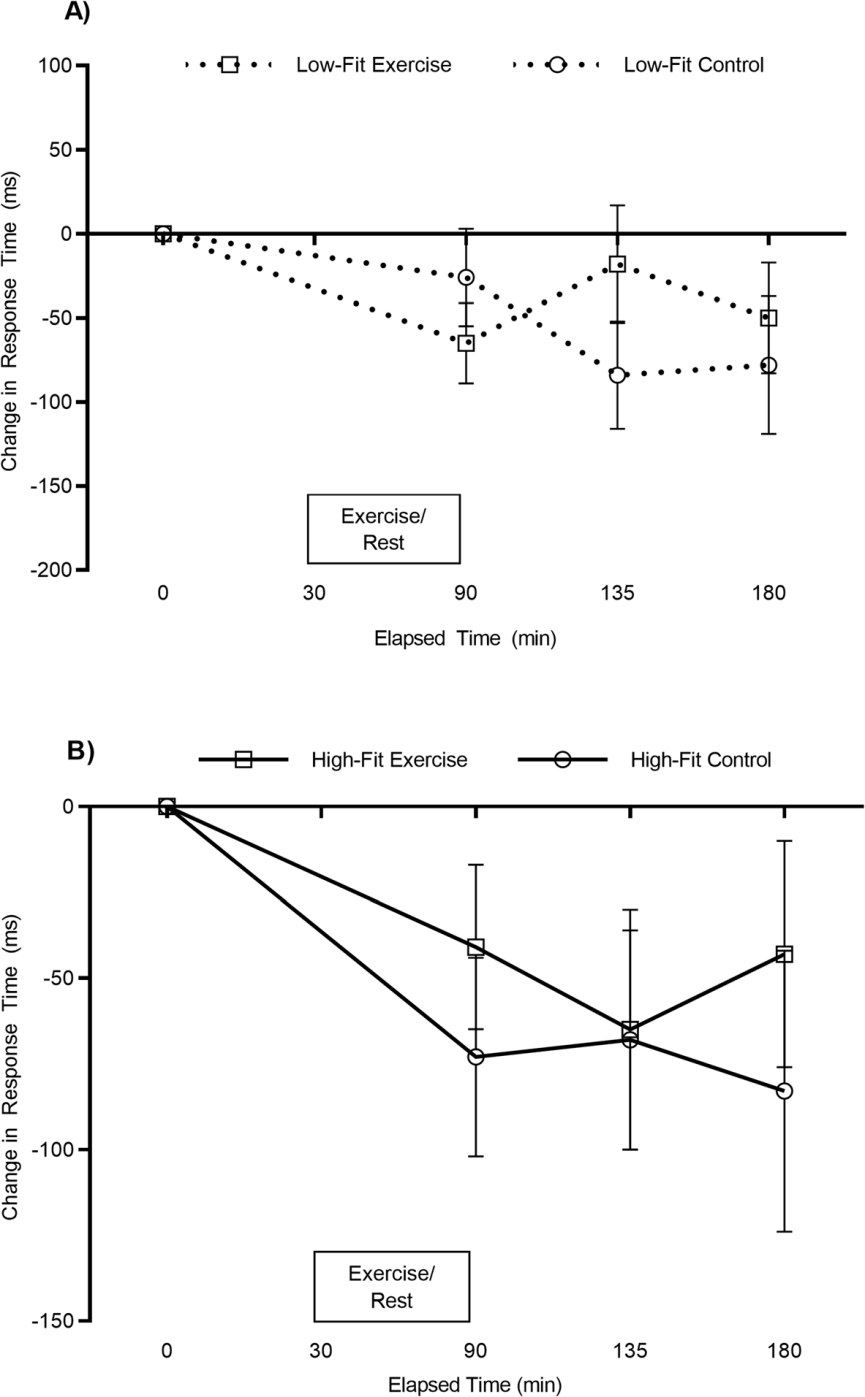
Congruent Stroop test response times across the exercise and resting trials for the low-fit (**a**) and high-fit (**b**) groups. Data are mean ± SEM and adjusted for waist circumference

Overall response times were quicker in the high-fit group compared to the low-fit group (main effect of fitness: high-fit; 719 ± 134 ms, low-fit; 794 ± 164 ms; *F*_(1, 5304)_ = 130.2, *p* < .001). Overall response times were similar between the exercise and resting trial (main effect of trial; *p* = .363) and became quicker across the course of the day (main effect of time; *F*_(3, 5304)_ = 18.5, *p* < .001). The pattern of change between the exercise and resting trial was similar (trial by time interaction: *p* = .373). The pattern of change across the exercise and resting trials was, however, different between the high- and low-fit groups (trial by time by fitness interaction; *F*_(3, 5304)_ = 4.8, *p* = .002; Fig. 1). A separate ANOVA revealed a difference in the pattern of change for the low-fit participants (trial by time interaction; *F*_(3, 2619)_ = 4.10, *p* = .007, Fig. 1a). Specifically, response times were quicker 45 min following seated rest compared to 45 min post-exercise (*p* = .045). However, in the high-fit participants, response times were similar across the morning between the exercise and resting trials (trial by time interaction; *p* = .231; Fig. 1b).

##### Incongruent Level

Overall response times were quicker in the high-fit group compared to low-fit (main effect of fitness; high-fit; 960 ± 209 ms, low-fit; 1084 ± 243 ms; *F*_(1, 10,668)_ = 317.1, *p* < .001). Overall response times were similar between the exercise and resting trial (main effect of trial; *p* = .994) and became quicker across the course of the day (main effect of time; *F*_(3. 10,668)_ = 22.4, *p* < .001). The pattern of change was similar between the exercise and resting trial (trial by time interaction: *p* = .204), as was the pattern of change between the high- and low-fit groups (trial by time by fitness interaction; *p* = .099).

##### Accuracy

**Congruent Level**

Overall accuracy was similar between the high- and low-fit groups (main effect of fitness; *p* = .316). Accuracy was also similar between the exercise and resting trial (main effect of trial; *p* = .324) and across the day (main effect of time; *p* = .409). The pattern of change across the day was similar between the exercise and resting trial (trial by time interaction; *p* = .428), as was the pattern of change between the high- and low-fit groups (trial by time by fitness interaction; *p* = .425). **Incongruent Level**

Overall accuracy was similar between the high- and low-fit groups (main effect of fitness; *p* = .317). Accuracy was also similar between the exercise and resting trial (main effect of trial; *p* = .317) and across the day (main effect of time; *p* = .410). The pattern of change across the day was similar between the exercise and resting trial (trial by time interaction; *p* = .410), as was the pattern of change between the high- and low-fit group (trial by time by fitness interaction; *p* = .413).

#### Sternberg paradigm

##### Response times

**One-item**

**Fig. 2:**
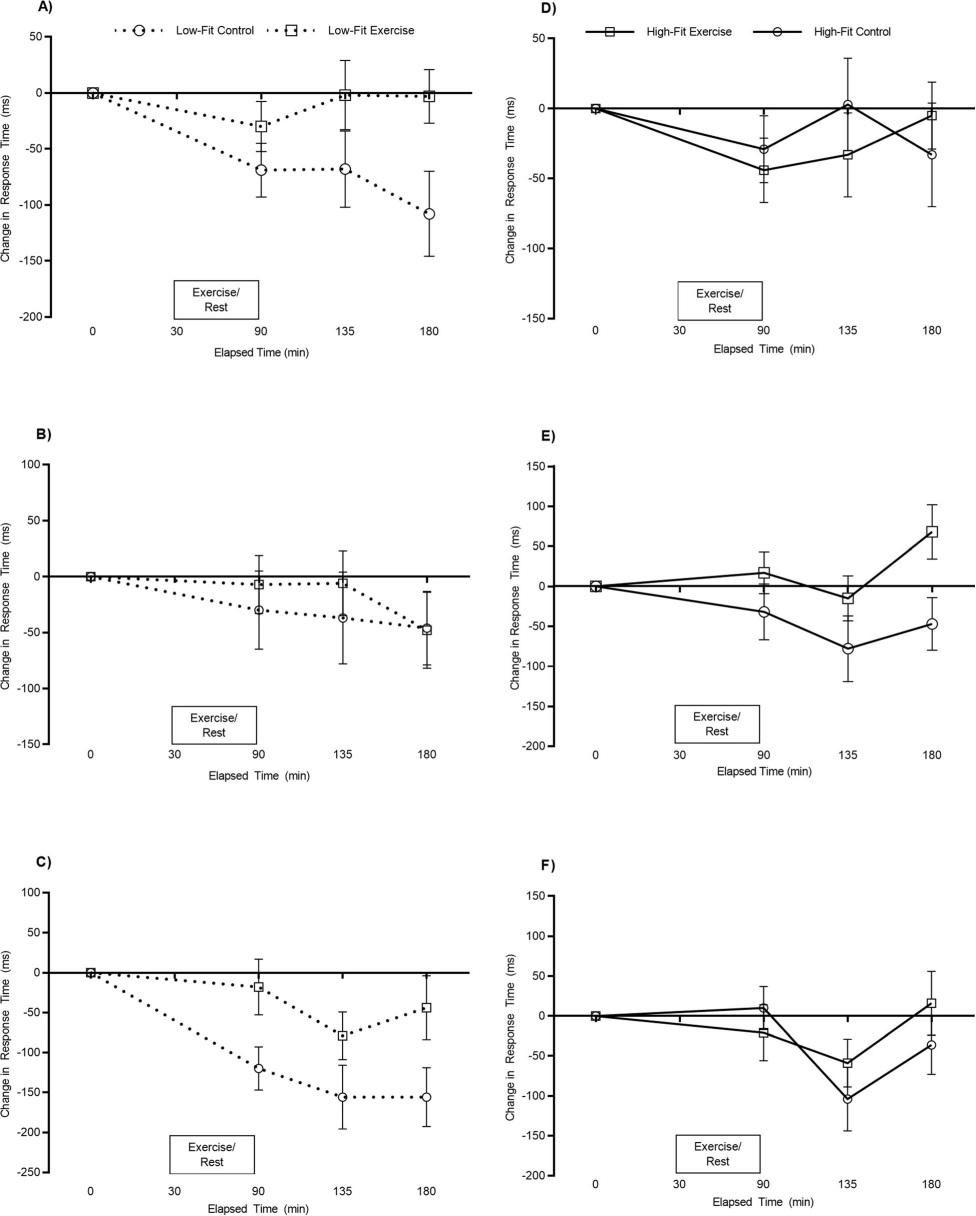
Response times, split by fitness group, across the exercise and resting trials for the One Item (**a** & **d**), Three Item (**b** & **e**) and Five Item (**c** & **f**) levels of the Sternberg Paradigm. Data are mean ± SEM and adjusted for waist circumference

Overall response times were quicker in the high-fit group compared to their low-fit counterparts (main effect of fitness; high-fit; 496 ± 91 ms, low-fit; 529 ± 124 ms, *F*_(1, 4372)_ = 44.1, *p* < .001). However, response times were similar between the exercise and resting trials (main effect of trial; *p* = .639) but became quicker across the course of the day (main effect of time; *F*_(3, 4372)_ = 11.8, *p* < .001). The pattern of change in response times was different between the exercise and resting trial (trial by time interaction: *F*_(3, 4372)_ = 9.2, *p* < .001) and furthermore, the pattern of change between the exercise and resting trial was different between the high- and low-fit participants (trial by time by fitness interaction; *F*_(3, 4372)_ = 4.2, *p* = .006). A separate ANOVA revealed a difference in the pattern of change for high-fit participants (trial by time interaction; *F*_(3, 2225)_ = 3.0, *p* = .030, Fig. 2d). Specifically, response times were quicker 45 min post-exercise, when compared to 45 min seated rest (*p* = .022). A separate ANOVA also revealed a difference in the pattern of change for low-fit participants (trial by time interaction; *F*_(3, 2147)_ = 9.7, *p* < .001, Fig. 2a). Specifically, low-fit participants were quicker 90 min following seated rest, when compared to 90 min post-exercise (*p* = .020).

**Three-item**

Overall response times were quicker in the high-fit group compared to low-fit (main effect of fitness; high-fit: 628 ± 115 ms, low-fit: 703 ± 160 ms, *F*_(1, 8725)_ = 184.4, *p* < .001). However, response times were similar between the exercise and resting trials (main effect of trial; *p* = .327) but became quicker across the course of the day (main effect of time; *F*_(3, 8725)_ = 7.5, *p* < .001). The pattern of change was different between the exercise and resting trials (trial by time interaction: *F*_(3, 8725)_ = 2.7, *p* = .042), as was the pattern of change across the day between the high- and low-fit groups (trial by time by fitness interaction; *F*_(3, 8725)_ = 3.9, *p* = .009). A separate ANOVA revealed a similar pattern of change between the exercise and resting trial for the low-fit participants (trial by time interaction; *p* = .390, Fig. 2b), yet there was a difference in the pattern of change for the high-fit participants (trial by time interaction; *F*_(3, 4388)_ = 6.5, *p* = < .001, Fig. 2e). Specifically, response times were quicker 90 min following seated rest, when compared to 90 min post-exercise (*p* < .001).

**Five-item**

Overall response times were also quicker in the high-fit group compared to low-fit on the five item level of the Sternberg paradigm (main effect of fitness; high-fit: 761 ± 151 ms, low-fit: 834 ± 207 ms, *F*_(1, 8236)_ = 99.8, *p* < .001). Overall response times were quicker in the control trial compared to the exercise trial (main effect of trial; exercise: 803 ± 168 ms, control: 791 ± 200 ms, *F*_(1, 8236)_ = 4.0, *p* = .046) and became quicker over the course of the day (main effect of time; *F*_(3, 8236)_ = 27.1, *p* < .001). The pattern of change was different between the exercise and resting trial (trial by time interaction: *F*_(3, 8236)_ = 5.6, *p* < .001) and furthermore, the pattern of change across the day was different between the high- and low-fit groups (trial by time by fitness interaction; *F*_(3, 8236)_ = 4.6, *p* = .003). A separate ANOVA revealed a difference in the pattern of change for the low-fit participants (trial by time interaction; *F*_(3, 4041)_ = 3.4, *p* = .018, Fig. 2c). Specifically, response times were slower immediately post-exercise compared to immediately after seated rest (*p* = .012) and slower 90 min post-exercise compared to 90 min following seated rest (*p* = .033). A separate ANOVA also revealed a difference in the pattern of change for the high-fit participants (trial by time interaction; *F*_(3, 4195)_ = 7.7, *p* < .001, Fig. 2f). Specifically, response times were quicker 45 min following seated rest compared to 45 min post-exercise (*p* = .003).

##### Accuracy

**One item**

Overall accuracy was similar between the high- and low-fit groups (main effect of fitness; *p* = .314), similar between the exercise and resting control trials (main effect of trial; *p* = .314). and similar across the course of the day (main effect of time; *p* = .398). The pattern of change across the day was similar between the exercise and resting trial (trial by time interaction; *p* = .396) as was the pattern of change between the high- and low-fit participants (trial by time by fitness interaction; *p* = .399).

**Three-item**

Overall accuracy was similar between the high- and low-fit groups (main effect of fitness; *p* = .315), similar between the exercise and resting trials (main effect of trial; *p* = .317) and similar across the course of the day (main effect of time; *p* = .398). The pattern of change across the day was similar between the exercise and resting trial (trial by time interaction; *p* = .394), as was the pattern of change between the high- and low-fit groups (trial by time by fitness interaction; *p* = .390).

**Five-item**

Overall accuracy was similar between the high- and low-fit groups (main effect of fitness; *p* = .321), similar between the exercise and resting trials (main effect of trial; *p* = .316) and similar across the course of the day (main effect of time; *p* = .404). The pattern of change across the day was similar between the exercise and resting trial (trial by time interaction; *p* = .400), as was the pattern of change between the high- and low-fit groups (trial by time by fitness interaction; *p* = .412).

### Brain derived Neurotrophic factor

Serum BDNF concentration was similar between the high- and low-fit groups (main effect of fitness; high-fit: 27.1 ± 6.8 ng·ml^− 1^, low-fit: 29.1 ± 7.8 ng·ml^− 1^, *p* = .210). Serum BDNF concentrations were also similar between the exercise and resting trial (main effect of trial; *p* = .082) and were also similar across the course of the day (main effect of time; *p* = .085). The pattern of change was similar between the exercise and resting trial (trial by time interaction; *p* = .167), as was the pattern of change between the high- and low-fit groups (trial by time by fitness interaction; *p* = .704, Table 4).

**Table 4:** Serum BDNF concentrations (ng·ml^− 1^) across the course of the resting and exercise trials, for the high- and low-fitness groups, as well as the group overall. Data are mean ± SD

Group	Control Trial					Exercise Trial				
	Pre-rest	Immediately post-rest	30 min post-rest	60 min post-rest	120 min post-rest	Pre-exercise	Immediately post-exercise	30 min post-exercise	60 min post-exercise	120 min post-exercise
Low-Fit	28.7 ± 6.3	29.2 ± 7.4	28.8 ± 6.2	31.1 ± 6.0	30.2 ± 8.2	28.1 ± 5.5	31.0 ± 10.6	28.4 ± 9.2	27.2 ± 8.2	28.5 ± 9.9
High-Fit	26.1 ± 5.0	26.3 ± 8.4	28.1 ± 6.7	29.3 ± 6.3	31.1 ± 7.3	23.4 ± 4.3	26.3 ± 7.2	26.8 ± 6.8	27.0 ± 5.9	26.3 ± 7.5
Overall	27.4 ± 5.8	27.8 ± 8.0	28.5 ± 6.3	30.2 ± 6.1	30.6 ± 7.7	25.8 ± 5.4	28.6 ± 9.2	27.6 ± 8.0	27.1 ± 7.1	27.4 ± 8.7

## Discussion

The findings of the present study show that acute Football activity did not influence subsequent information processing, inhibitory control and working memory response times for this group of adolescents overall. However, response times for the high-fit group were quicker across all levels of cognitive tasks, compared to the low-fit group. When considering the moderating role of fitness on the acute responses to exercise, 60 min of Football was beneficial for working memory in the high-fit group, whereas working memory tended to be unaffected by exercise in the low-fit group. The present study is also the first to measure the time course of BDNF post-exercise in an adolescent population, with serum BDNF unaffected by acute Football activity and fitness.

The current study demonstrates that response times, during information processing, inhibitory control and working memory tasks, are quicker in adolescents with a higher physical fitness, when compared to their low-fit counterparts. This is in support of recent meta-analyses in children and adolescents demonstrating that chronic exercise interventions, which aim to improve physical fitness, lead to improvements in cognitive function [49, 50]. The findings of the present study extends previous cross-sectional findings in children [26–29] and adults [25, 32] to three distinct domains of cognitive function (information processing, inhibitory control and working memory) in adolescents. Response times were consistently quicker in the high-fit group across the congruent and incongruent levels of the Stroop Task, as well as across all three levels of the Sternberg Paradigm, compared to the low-fit group. This enhanced cognition in high-fit adolescents may explain the improved academic performance in high-fit young people that has previously been reported [28, 51, 52] The findings of the current study, along with previous work, highlight the importance of high levels of physical fitness for cognitive function and academic achievement in children and adolescents.

The current study also demonstrates that the acute benefits to working memory following exercise were exclusive to the high-fit group only. This is an important finding, given that physical fitness is suggested as a key moderator of the exercise-cognition relationship [1, 4], yet there are few empirical studies directly investigating this, especially in adolescents. Recent work has investigated this through a 60 min Basketball session [16] and a 20 min bout of cycling [11]. Even though the modality and duration of exercise are vastly different, both studies concluded that the improvement in cognition, following an acute bout of exercise, was enhanced in those considered high-fit; in line with the findings of the present study. An explanation for this may be the differences in relative exercise intensity during the Football activity, with the low-fit group working at a higher relative exercise intensity (~ 80% HRmax) compared to the higher fit children (~ 70% HRmax). It is possible that for the low-fit children the exercise was of too high an intensity and thus too demanding. A recent review suggests that enhancements in cognitive function, following exercise, tend to occur under moderate intensities with attenuated effects under light- and high-intensities; which is consistent with an inverted-U theory [4]. An additional explanation might be under the transient hypofrontality hypothesis, whereby neural activity in the brain – mainly the prefrontal cortex – is reduced as a result of very high-intensity exercise [53].

The present study also provides novel evidence regarding the effects of Football on subsequent cognitive function (particularly working memory) in adolescents, with only one previous studying investigating the acute effects of Football [24]. The majority of previous work in adolescents has used traditional forms of exercise; such as continuous running [6, 7, 19, 20], walking [8] and cycling [9, 11, 12]. Whilst traditional exercise protocols are easy to control in a laboratory setting, they do not necessarily reflect the habitual activity patterns of young people [21]. The use of Football may provide an attractive model; viable for adolescents and thus has real-world applicability. Whilst the cognitive benefits following Football were exclusive to high-fit individuals and the domain of working memory in the present study, there was also no evidence of a decline in performance for the low-fit participants because of exercise. This suggests that games-based exercise, such as Football, can still be a valid mode of activity for young people, given the known health benefits [54], the popularity [46] and the ease of access to the equipment needed.

The present study is the first to examine the time-course of circulating BDNF in adolescents following an acute bout of exercise. This is an important knowledge gap, given the potential mediating role of BDNF in the exercise-cognition relationship [31, 33, 55] and the transient nature of improvements seen in cognitive function following exercise. Whilst peripheral BDNF increases immediately after acute bouts of exercise in adults [55, 56], data from the current study did not provide evidence of this effect in adolescents. The post-exercise increase in BDNF is positively associated with the intensity and duration of the exercise bout [55]. The exercise bout in the current study was of a sufficient duration, however the intensity may not have been sufficient enough to elicit increases in BDNF post-exercise. The present study did however demonstrate cognitive improvements post-exercise, despite the lack of change in peripheral BDNF. This may be explained by the fact that central BDNF (in the brain) was not measured in the present study, due to the constraints of such an assessment in adolescents, and arguably central BDNF is more important for the cognitive benefits of exercise. The improvements in response times seen in the present study, without any noticeable change in peripheral BDNF, may be explained by this or suggest that another mechanism is mediating these cognitive benefits.

A potential limitation of the present study is that the socioeconomic status of the participants was not accounted for. It has been reported that socioeconomic status is implicated in the development of attentional processes in young children [57] and executive function throughout childhood and adolescence [58]. However, there is evidence suggesting that a lower socioeconomic status is associated with lower levels of physical activity [59] and physical fitness [60] in adolescents; which suggests that the effect of socioeconomic status on cognitive function may, in part, be mediated through physical activity and physical fitness. The relationship between physical fitness and cognitive function in the present study is cross-sectional and thus, causation cannot be attributed. However, this is still an important finding as this relationship was evident across all test levels for both the Stroop test and the Sternberg paradigm. Whilst the number of trials used in both the Stroop and Sternberg tests could be seen as a limitation, it was necessary to reduce the amount in order to facilitate the use of both tests within a realistic timeframe. The choice of control condition (seated rest) in the present study could also be seen as a potential limitation, particularly as the exercise session included both physical and cognitive elements. However, it would be difficult to match the social interactions of the exercise session and the use of such a control condition also offers ecological validity. A further potential limitation is the measurement of global response time (rather than reaction time and movement time separately), due to the practicalities of conducting such measurements in field-based studies of this nature.

## Conclusion

Overall, the findings of the present study show that high-fit participants performed better across tests of information processing, inhibitory control and all levels of working memory tasks compared to the low-fit group. In addition, the current study also provides novel evidence supporting physical fitness as a moderator of the exercise-cognition relationship. In particular, working memory was improved in the high-fit group 45 min post-exercise, whereas it was unaffected in the low-fit group. The present study also provides novel evidence that a 60 min bout of Football did not alter peripheral BDNF concentration in an adolescent population, nor was BDNF affected by physical fitness. Overall, these findings suggest that physical fitness is an important determinant of cognitive performance in adolescents; and that acute bouts of exercise, appropriate to the fitness levels of the young people, can also enhance subsequent cognition. This suggests that opportunities for exercise within the school day must be appropriate for the young people and, importantly, that a ‘one size fits all’ approach will not elicit cognitive benefits for all young people.

## Supplementary information

**Additional file 1.** A visual representation of the testing procedures for the Stroop Test and Sternberg Paradigm. Description of Data: These figures show exactly what participants saw on their laptop screens and outlines the instructions that were provided to them; i.e. what to do, how to do it. Copyright: these images were developed by the research team and have no copyright associated with them.

**Additional file 2. **Main effects (Trial, time and fitness), two-way interactions (trial by time) and three-way interactions (trial by time by fitness) for response times and accuracy across all test levels of the Stroop and Sternberg tests. Description of data: This table summarises the statistical outputs (*p* values) for the cognitive function tests to facilitate interpretation.

## Data Availability

The dataset used for the current study are available from the corresponding author upon request.

## Abbreviations

BDNF: Brain-derived Neurotrophic Factor
GPS: Global Positioning System
HR: Heart Rate
HRmax: Maximum Heart Rate
MSFT: Multi-stage Fitness Test
