# Supplementary material for: Effect of football activity and physical fitness on information processing, inhibitory control and working memory in adolescents
Source: BMC Public Health. 2020 Sep 14;20:1398. doi: 10.1186/s12889-020-09484-w (PMC7488749; doi:10.1186/s12889-020-09484-w)
Supplement: Supplementary file 1 — Additional file 1. A visual representation of the testing procedures for the Stroop Test and Sternberg Paradigm. Description of Data: These figures show exactly what participants saw on their laptop screens and outlines the instructions that were provided to them; i.e. what to do, how to do it. Copyright: these images were developed by the research team and have no copyright associated with them. [file 12889_2020_9484_MOESM1_ESM.pdf]

Stroop Test – Congruent Level

1A)

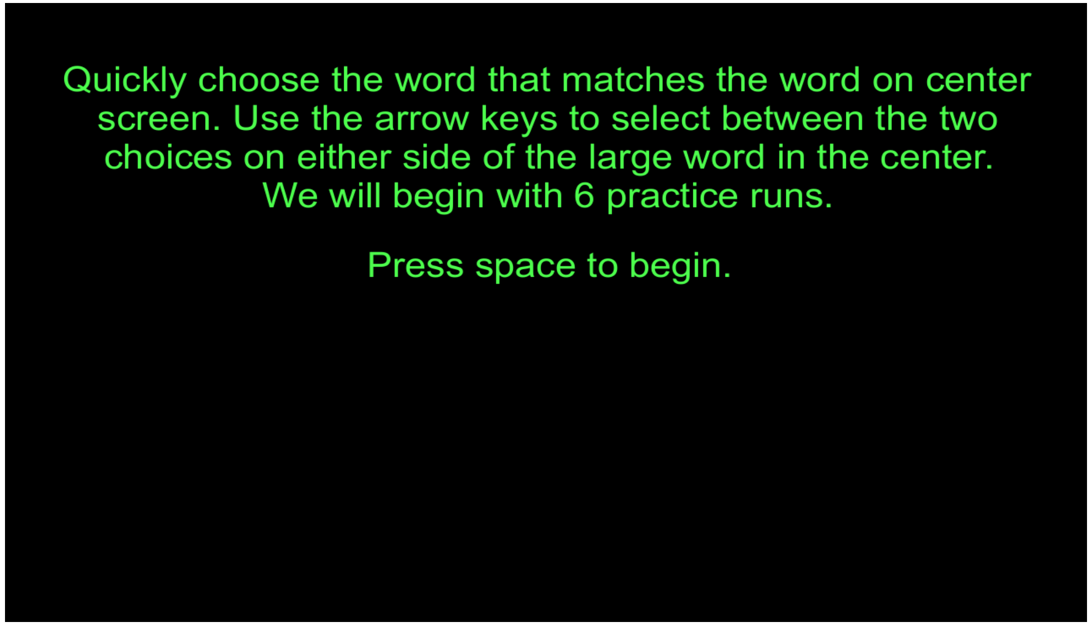

On-screen instructions that were provided at the start of each test. These informed participants that they had 6 practice runs (with feedback) before commencing with the real test (no feedback).

1B)

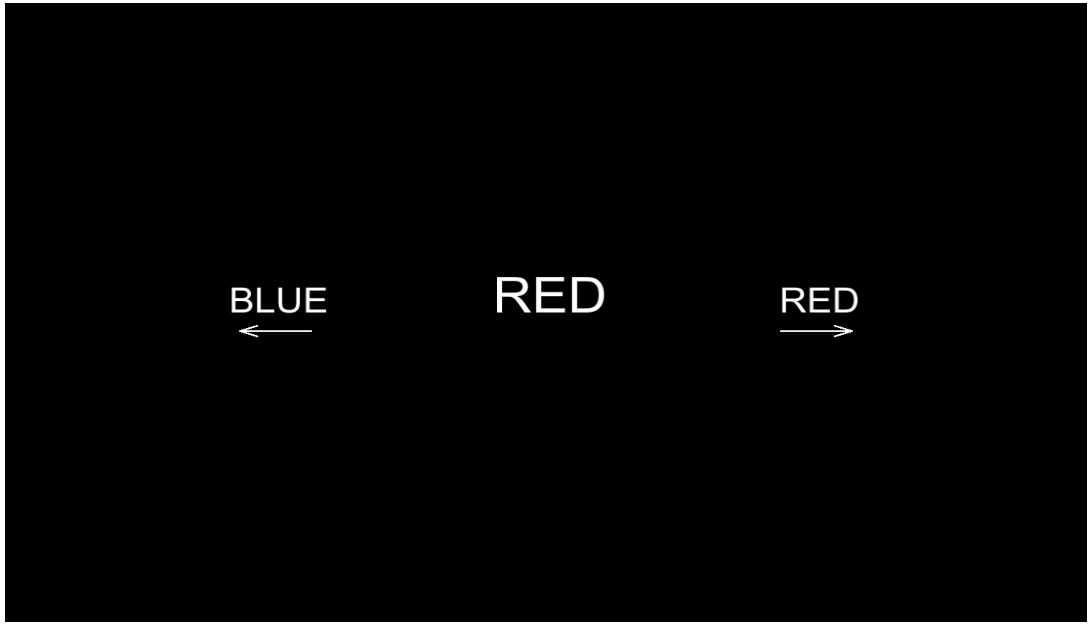

Visual representation of the stimuli presented during the congruent level. The middle word (RED) is the target. Participants were instructed to identify the target using either the right (middle finger) or left arrows (index finger) on the laptop keys.

For this particular example, the correct response is the right arrow. Participants were instructed to respond as quickly as possible, whilst selecting the correct response.

Stroop Test – Incongruent Level

C)

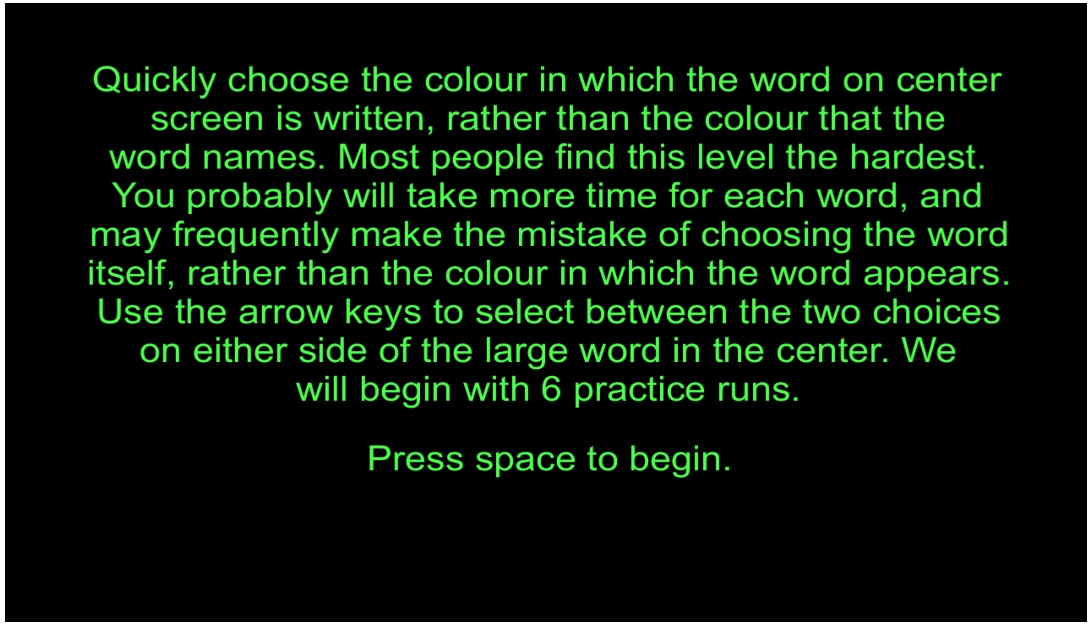

On-screen instructions that were provided at the start of each test. These informed participants that they had 6 practice runs (with feedback) before commencing with the real test (no feedback).

D)

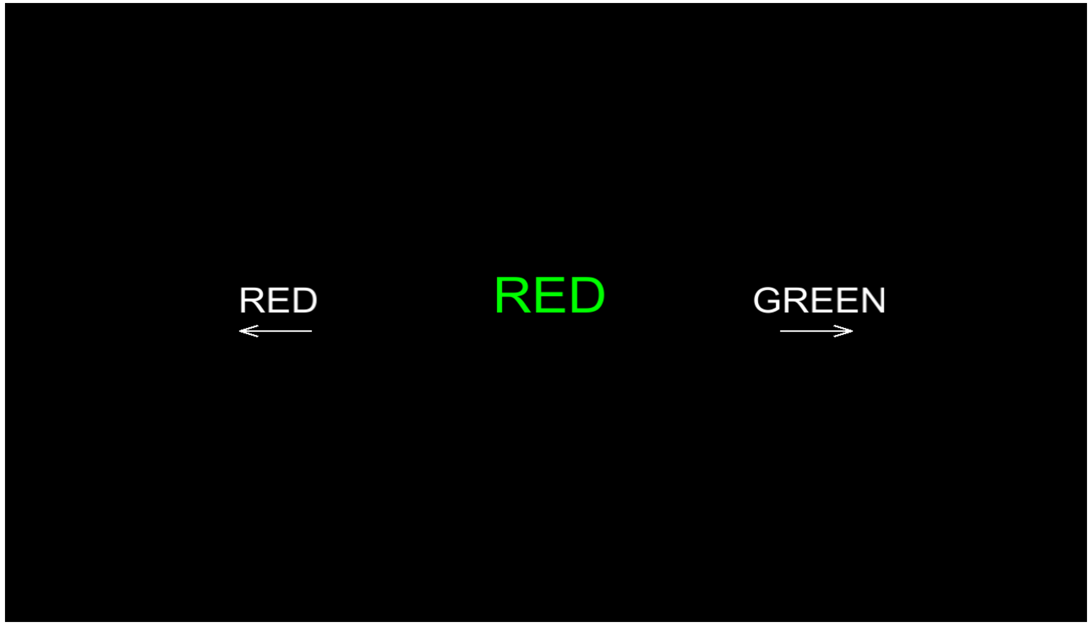

Visual representation of the stimuli presented during the incongruent level. The *colour ink* of the middle word (GREEN) is the target. Participants were instructed to identify the target using either the right (middle finger) or left arrows (index finger) on the laptop keys.

For this particular example, the correct response is the right arrow. Participants were instructed to respond as quickly as possible, whilst selecting the correct response.

Sternberg Paradigm – All levels

N.B. The example here is for the five-letter level. The same principles apply to the other levels, only the number of stimuli to remember differs (i.e. 1 item or 3 items)

E)

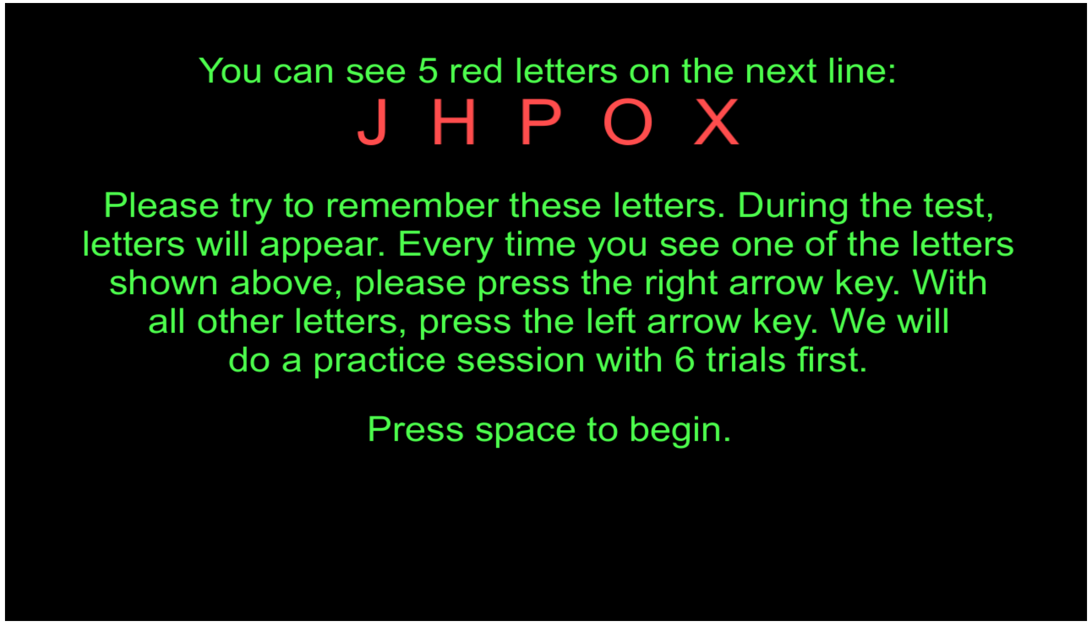

On-screen instructions that were provided at the start of each test. These informed participants that they had 6 practice runs (with feedback) before commencing with the real test (no feedback).

F)

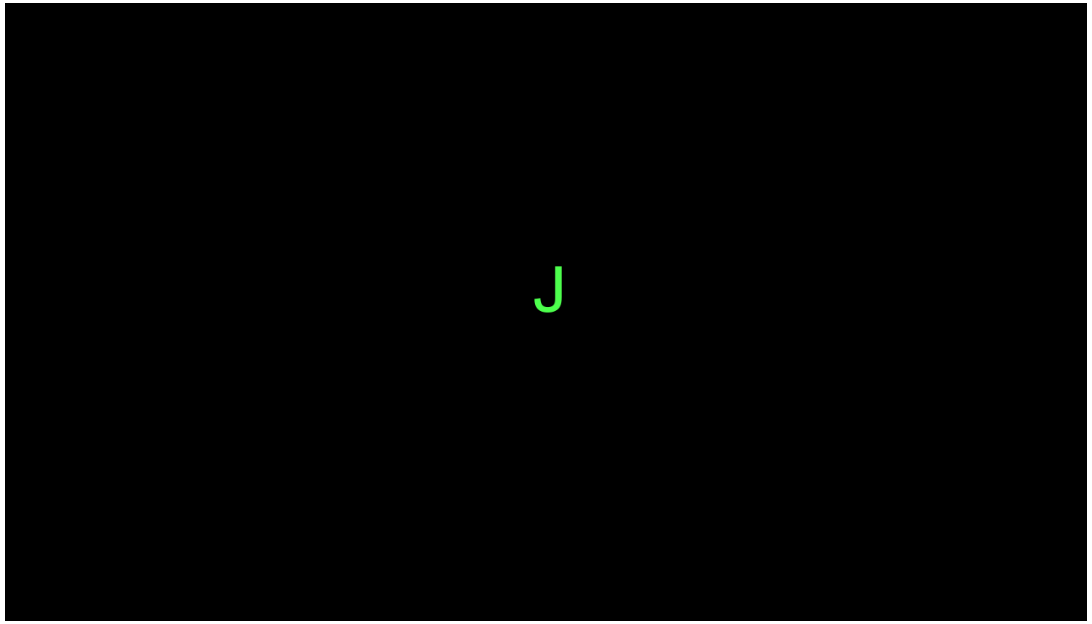

Visual representation of the stimuli presented during the Sternberg Paradigm. Participants were required to press the right arrow (middle finger), if the on screen item was their target stimuli, or the left arrow (index finger) if it was any other item.

For this particular example, as “J” is a target item (See E), the correct response would be the right arrow. Participants were instructed to respond as quickly as possible, whilst selecting the correct response.
