# Supplementary material for: Effect of football activity and physical fitness on information processing, inhibitory control and working memory in adolescents
Source: BMC Public Health. 2020 Sep 14;20:1398. doi: 10.1186/s12889-020-09484-w (PMC7488749; doi:10.1186/s12889-020-09484-w)
Supplement: Supplementary file 2 — Additional file 2. Main effects (Trial, time and fitness), two-way interactions (trial by time) and three-way interactions (trial by time by fitness) for response times and accuracy across all test levels of the Stroop and Sternberg tests. Description of data: This table summarises the statistical outputs (p values) for the cognitive function tests to facilitate interpretation. [file 12889_2020_9484_MOESM2_ESM.docx]

| Test | Test Level | Variable | Main Effects | | | Interactions | | Post-Hoc ANOVA | |
| --- | --- | --- | --- | --- | --- | --- | --- | --- | --- |
|  |  |  | Trial | Time | Fitness | Trial*Time | Trial*Time*Fitness | Low-Fit | High-Fit |
| Stroop Test | Congruent | Response Time | .363 | <.001 | <.001 | .373 | .002 | .007 | .231 |
|  |  | Accuracy | .324 | .409 | .316 | .428 | .425 | - | - |
|  | Incongruent | Response Time | .994 | <.001 | <.001 | .204 | .099 | - | - |
|  |  | Accuracy | .317 | .410 | .317 | .410 | .413 | - | - |
| Sternberg Paradigm | One Item | Response Time | .639 | <.001 | <.001 | <.001 | .006 | <.001 | .030 |
|  |  | Accuracy | .314 | .398 | .314 | .396 | .399 | - | - |
|  | Three Item | Response Time | .327 | <.001 | <.001 | .042 | .009 | .390 | <.001 |
|  |  | Accuracy | .317 | .398 | .315 | .394 | .390 | - | - |
|  | Five Item | Response Time | .046 | <.001 | <.001 | <.001 | .003 | .018 | <.001 |
|  |  | Accuracy | .316 | .404 | .321 | .400 | .412 | - | - |

Supplementary Table 1: An overview of the results of the statistical analyses performed

Note: post-hoc ANOVAs were only run where a significant three-way (trial * time * fitness) interaction existed.
